# Impact of Albumin and Omeprazole on Steady-State Population Pharmacokinetics of Voriconazole and Development of a Voriconazole Dosing Optimization Model in Thai Patients with Hematologic Diseases

**DOI:** 10.3390/antibiotics9090574

**Published:** 2020-09-03

**Authors:** Buddharat Khan-asa, Baralee Punyawudho, Noppaket Singkham, Piyawat Chaivichacharn, Ekapun Karoopongse, Preecha Montakantikul, Methee Chayakulkeeree

**Affiliations:** 1 Division of Clinical Pharmacy, Department of Pharmacy, Faculty of Pharmacy, Mahidol University, Bangkok 10400, Thailand; bhudharat.kha@mahidol.ac.th; 2 Inpatient Pharmacy Department, Pharmacy Department, Faculty of Medicine Siriraj Hospital, Mahidol University, Bangkok 10700, Thailand; 3 Department of Pharmaceutical Care, Faculty of Pharmacy, Chiang Mai University, Chiang Mai 50200, Thailand; baralee.p@cmu.ac.th (B.P.); watrxbuu@gmail.com (P.C.); 4 School of Pharmaceutical Sciences, University of Phayao, Phayao 56000, Thailand; noppaket.si@up.ac.th; 5 Division of Hematology, Department of Medicine, Faculty of Medicine Siriraj Hospital, Mahidol University, Bangkok 10700, Thailand; sikkf@mahidol.ac.th; 6 Division of Infectious Diseases and Tropical Medicine, Department of Medicine, Faculty of Medicine Siriraj Hospital, Mahidol University, Bangkok 10700, Thailand

**Keywords:** population pharmacokinetics, voriconazole, albumin, omeprazole, Thai patients

## Abstract

This study aimed to identify factors that significantly influence the pharmacokinetics of voriconazole in Thai adults with hematologic diseases, and to determine optimal voriconazole dosing regimens. Blood samples were collected at steady state in 65 patients (237 concentrations) who were taking voriconazole to prevent or treat invasive aspergillosis. The data were analyzed using a nonlinear mixed-effects modeling approach. Monte Carlo simulation was applied to optimize dosage regimens. Data were fitted with the one-compartment model with first-order absorption and elimination. The apparent oral clearance (CL/F) was 3.43 L/h, the apparent volume of distribution (V/F) was 47.6 L, and the absorption rate constant (Ka) was fixed at 1.1 h^−1^. Albumin and omeprazole ≥ 40 mg/day were found to significantly influence CL/F. The simulation produced the following recommended maintenance doses of voriconazole: 50, 100, and 200 mg every 12 h for albumin levels of 1.5–3, 3.01–4, and 4.01–4.5 g/dL, respectively, in patients who receive omeprazole ≤ 20 mg/day. Patients who receive omeprazole ≥ 40 mg/day and who have serum albumin level 1.5–3 and 3.01–4.5 g/dL should receive voriconazole 50 and 100 mg, every 12 h, respectively. Albumin level and omeprazole dosage should be carefully considered when determining the appropriate dosage of voriconazole in Thai patients.

## 1. Introduction

Patients with hematologic diseases that receive chemotherapy are at high risk for invasive fungal infection, which is a severe illness with high mortality [1,2,3]. Voriconazole is a broad-spectrum triazole antifungal that is highly effective against a wide range of yeasts and filamentous fungi [4]. In the treatment of invasive aspergillosis, voriconazole was shown to improve survival and have less side effects as compared with a previous standard treatment with amphotericin B deoxycholate [5]. Voriconazole has been approved for adult and pediatric patients with various invasive fungal infections, including invasive aspergillosis (a first-line drug), candidemia in non-neutropenic patients, disseminated infections caused by *Candida* spp., esophageal candidiasis, and scedosporiosis and fusariosis in patients refractory or intolerant of other antifungal agents [1,2,3,4,6]. Numerous studies have reported that voriconazole can prevent invasive fungal infections in immunocompromised patients [2,3,6,7,8].

Many practice guidelines recommend therapeutic drug monitoring of voriconazole to guide dosing optimization. At steady state, trough concentrations of voriconazole greater than 1–2 mg/L have been associated with good clinical outcomes, whereas trough concentrations more than 5–6 mg/L have been related to neuropsychiatric toxicity [2,3,9,10]. Voriconazole exhibits nonlinear pharmacokinetics (PK), and it has high intra-variability and inter-variability among subjects [4,11], which makes it difficult to predict plasma concentrations for dose adjustment. Voriconazole is mainly metabolized by *CYP2C19* [4,11]. Therefore, high variability in the PK of voriconazole can be caused by *CYP2C19* polymorphism [12,13,14,15,16,17,18,19,20,21], and these gene polymorphisms cause variations in drug clearance. Clinical factors that have been reported to affect the PK of voriconazole include body weight [15,16,20,22,23], age [18,19], liver function [20], aspartate aminotransferase (AST) [14], direct bilirubin (DB) [24], and voriconazole taken concurrently with *CYP2C19* inducers (rifampin) [13,25], and inhibitors (proton pump inhibitors; PPIs) [17]. PPIs are commonly prescribed in patients with hematologic diseases to treat or prevent gastroduodenal adverse effects from chemotherapy, thrombocytopenia, and high-dose glucocorticoids [26,27]. Moreover, the majority of these patients were found to have hypoalbuminemia [28]. There is some evidence supporting the fact that low albumin levels can affect the plasma level of voriconazole [29]. However, there is no population PK study confirming this effect [12]. Population PK analysis is a strategy that can be employed to identify mean PK parameters and their variation, to identify significant covariates, and to establish the optimal dosage regimen of voriconazole [12,13,14,15,16,17,18,19,20,21,22,23,24,25].

Numerous population PK studies of voriconazole have been conducted. Those studies found that various factors affected PK parameters in patient populations that included organ transplantation, critically ill status, or malignancy. Moreover, race (i.e., Caucasian vs. Asian) can also contribute to different PK parameters. The prevalence of *CYP2C19* poor metabolizer has been reported to be about 15–20% in the Asian population [30]. Interestingly, we found some different factors that influence the clearance of voriconazole between Chinese [19] and Korean patients [20]. However, there are no studies that have investigated the population PK of voriconazole in Thai patients. Therefore, the aim of this study was to develop a population PK model to identify factors that significantly influence the PK of voriconazole in Thai patients with hematologic diseases. Our secondary objective was to develop a dosing optimization model using Monte Carlo simulation, and to identify significant covariates to determine optimal voriconazole regimens in this patient population.

## 2. Results

### 2.1. Patient Characteristics

Plasma voriconazole concentrations were collected from 65 subjects (237 concentrations), including 18 intensive PK subjects on the seventh day of voriconazole (126 concentrations), and 47 subjects from whom trough concentration was evaluated at different times during the seventh day to the 40th day of voriconazole (111 concentrations). The median voriconazole plasma concentration was 4.49 mg/L (interquartile range (IQR) 4–12 h) and blood samples were taken from 0.75–12 h post dose (median 11.5 h and IQR 4–12 h). All patients included in the study were Thai adults with hematologic diseases. The baseline demographic and clinical characteristics of the study patients are shown in Table 1.

### 2.2. Population PK Model Development

The PK profile of voriconazole can best be described using the one-compartment model with first-order absorption and elimination (see the model code in the Supplementary Materials). The two-compartment model did not adequately fit the data. Additionally, nonlinear (Michaelis–Menten) elimination did not improve the model fit. The apparent oral clearance (CL/F), the apparent volume of distribution (V/F), and the absorption rate constant (Ka) were estimated. The estimated Ka was not reliable, therefore, we decided to fixed it with a value from a previous population PK study (1.1 h^−1^) [25]. The sensitivity analysis of various Ka was shown in Table S1 in Supplementary Materials. The interindividual variability (IIV) of CL/F was described using an exponential model. The addition of IIV for V/F failed to fit the data, and estimates were near parameter boundaries. The objective function value (OFV) of allometric scaling was not significantly different from the base model. Thus, the variability in Ka and V/F was not estimated. The residual variability (RUV) was described using an additive model. The shrinkage value of CL/F was 11%, indicating enough data available for each patient to estimate the individual parameters reliably.

Covariate analysis showed that clearance was significantly affected by albumin and dose of omeprazole ≥ 40 mg/day. CYP2C19*2/*3 and sulfamethoxazole/trimethoprim were found to be significant only during forward selection. Age, gender, weight, *CYP2C19* genotypes or phenotypes, AST (aspartate aminotransferase), ALT (alanine aminotransferase), ALP (alkaline phosphatase), TB (total bilirubin), DB (direct bilirubin), and co-medication, including omeprazole 20 mg/day and hormones, were not found to be significant covariates.

As the only two significant covariates, albumin and omeprazole ≥ 40 mg/day were included in the final model. These factors resulted in a 15.4% drop in the IIV of CL/F from the base model, and a drop in the OFV of 22.657 points (*p* < 0.001). The equation used in the final model is shown, as follows:

CL/F (L/h) = 3.43 × [1 + 0.249 × (ALB − 3.2)] × [1 + (−0.306 × OME)]

where ALB is the plasma albumin (g/dL), and OME = 0 for patients receiving omeprazole 20 mg/day or less, and 1 for patients receiving a dose of omeprazole ≥ 40 mg/day.

### 2.3. Model Evaluation and Validation

**Figure 1:**
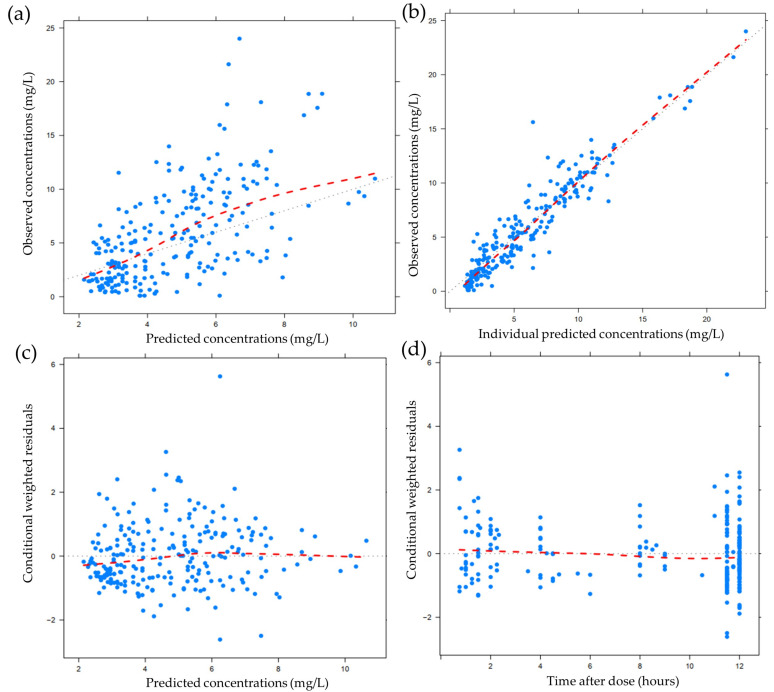
Goodness-of-fit (GOF) plots for the final model. (**a**) Population-predicted concentration (PRED) versus observed concentration; (**b**) Individual predicted concentration (PRED) versus observed concentration; (**c**) Conditional weighted residuals (CWRES) versus PRED; (**d**) CWRES versus time after dose; The red lines in (**a**) and (**b**) show regression lines, and in (**c**) and (**d**) they show the positive where CWRES equals 0.

Table 2 shows the analysis that compared the 1000 bootstraps with the final model. The parameter estimates obtained from bootstrap were similar to the NONMEM estimates, which demonstrates the stability of the parameter estimates from the final model. The goodness-of-fit (GOF) evaluation was performed by plotting the corresponding individual predictive values (IPRED) and population predictive values (PRED) with the observed values, as well as the time and PRED with conditional weighted residual errors (CWRES). These plots showed good scattering with all points located within ±4, and most points within ±2 (Figure 1).

**Figure 2:**
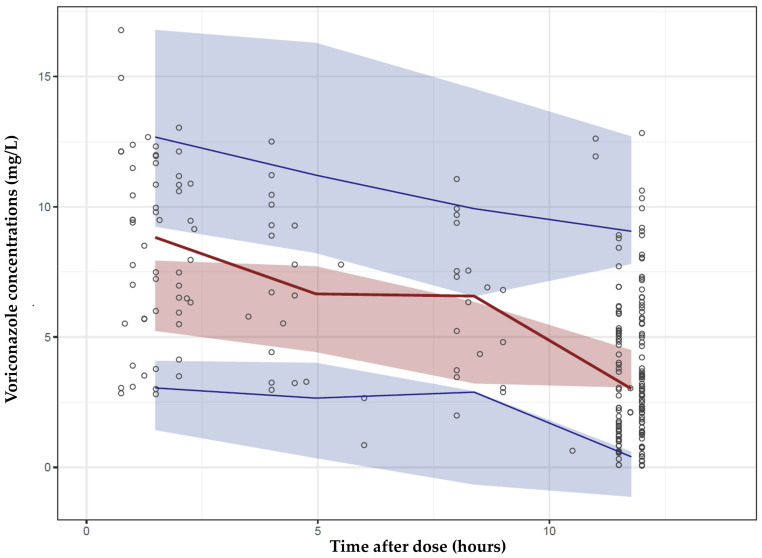
Prediction-corrected visual predictive check (pcVPC) plot of the final model. Open circles show observed plasma voriconazole concentrations. The red line is 50th percentile, and blue lines are the 5th and 95th percentiles, of the observed concentrations. Shaded areas are the 95% CIs of the corresponding model-predicted percentiles.

Prediction-corrected visual predictive check (pcVPC) and visual predictive check (VPC) were used to test the predictability of the final model. The results of pcVPC indicated that the final model had adequate predictive properties. Figure 2 shows the median, 5th percentile, and 95th percentile of the observed plasma concentrations that were included in the range of the 95% confidence interval (CI) of these percentiles for the simulated data. Those findings suggested the sufficiency of the model’s predictive power. Figure S1 in Supplementary Materials shows the traditional VPC plot.

### 2.4. Simulation

**Figure 3:**
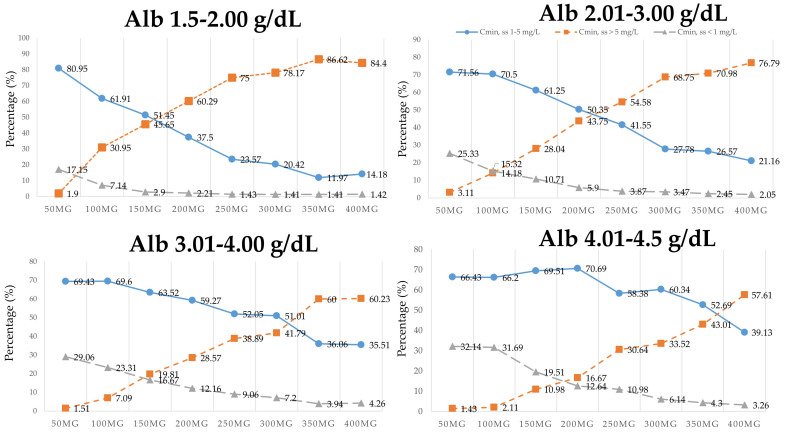
Simulated steady-state trough or minimal concentrations (C_min, ss_) in the non-omeprazole group or omeprazole 20 mg/day group stratified by plasma albumin (Alb) and a maintenance dose of voriconazole administration (50, 100, 150, 200, 250, 300, 350, and 400 mg orally every 12 h of voriconazole). The round, square, and triangle lines show the percentage that achieved a therapeutic range (C_min, ss_ = 1–5 mg/L), a toxic range (C_min, ss_ > 5 mg/L), and a sub-therapeutic range (C_min, ss_ < 1 mg/L), respectively.

**Figure 4:**
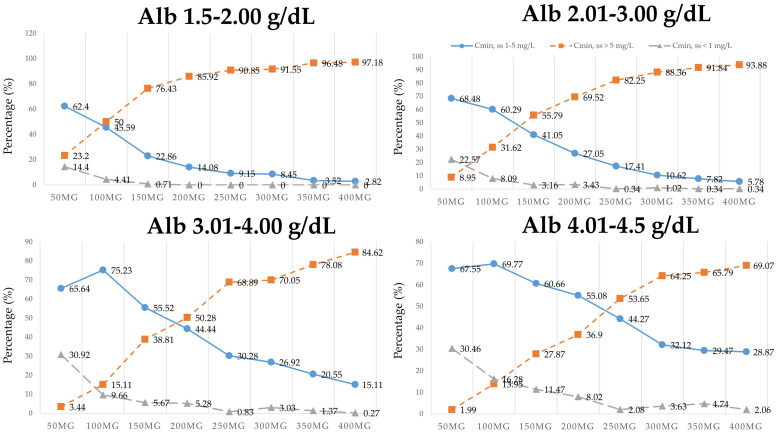
Simulated steady-state trough or minimal concentrations (C_min, ss_) in the omeprazole ≥ 40 mg/day group stratified by plasma albumin (Alb) and voriconazole dose administration (50, 100, 150, 200, 250, 300, 350, and 400 mg orally every 12 h of voriconazole). The round, square, and triangle lines show the percentage that achieved a therapeutic range (C_min, ss_ = 1–5 mg/L), a toxic range (C_min, ss_ > 5 mg/L), and a sub-therapeutic range (C_min, ss_ < 1 mg/L), respectively.

Monte Carlo simulations were performed using the final model to determine the optimal dosing regimens for Thai patients. Steady-state trough or minimal concentrations (C_min, ss_) of voriconazole were simulated using the final model that included the significant covariates for CL/F. Sixteen thousand (16,000) replicate C_min, ss_ of voriconazole using the dosage regimens of 50, 100, 150, 200, 250, 300, 350, and 400 mg twice daily (2000 replicates per group) were simulated for each covariate subgroup. The simulated C_min, ss_ were randomized according to various plasma albumin levels 1.5–4.5 g/dL (all plasma albumin levels ranged from 1.6 to 4.5 g/dL; median: 3.2 g/dL, IQR: 2.78–3.6 g/dL), and then these were classified into two omeprazole dosing groups as follows: 1000 replicates for non-omeprazole and omeprazole 20 mg/day, and 1000 replicates for omeprazole ≥ 40 mg/day. The simulated C_min, ss_ in different subgroups are shown in Figure 3 and Figure 4.

For example, in the group that included non-omeprazole or omeprazole 20 mg/day and albumin 1.5–2 g/dL, the maintenance dose of voriconazole, 150 mg orally every 12 h, yielded the percentage of C_min, ss_ of voriconazole that achieved a therapeutic range of voriconazole (1–5 mg/L) of 51.45%, whereas the percentage that achieved a toxic range (C_min, ss_ > 5 mg/L) was 45.65%, and the percentage that achieved a sub-therapeutic range (C_min, ss_ < 1 mg/L) was 2.90%. In the group of omeprazole ≥ 40 mg/day and albumin 1.5–2 g/dL, the maintenance dose of voriconazole, 150 mg orally every 12 h, yielded a percentage of C_min, ss_ of voriconazole that achieved a therapeutic range of voriconazole (1–5 mg/L) of 22.86%, while the percentage that achieved a toxic range (C_min, ss_ > 5 mg/L) was 76.43%, and the percentages that achieved a sub-therapeutic range (C_min, ss_ < 1 mg/L) was 0.71%.

## 3. Discussion

To our knowledge, this is the first population PK study of voriconazole in Thai adults with hematologic diseases. We investigated factors that influence PK parameters of voriconazole, and we performed Monte Carlo simulation to determine dosing recommendations for Thai patients. In the present study, first, we established the lower albumin level and the higher dose of omeprazole ≥ 40 mg/day that were able to describe the variability of CL/F. In addition, optimal voriconazole dosage recommendations were established according to these covariates.

The model which best described the PK profile of voriconazole was the one-compartment model with first-order absorption and elimination, which was consistent with previous studies [14,16,18,19,21,25,31]. However, Mangal et al. characterized PK with one compartment with first-order absorption and Michaelis–Menten elimination [17]. Alternatively, a study by Han et al. described the voriconazole data using the two-compartment model with first-order absorption and elimination [22], whereas Dalton et al. [13], Liu et al. [15], and Friberg et al. [23] employed a two-compartment model with first-order absorption, a lag time, a mixed linear approach, and Michaelis–Menten elimination. We explored the Michaelis–Menten elimination and two-compartment models, but these models did not fit our data. The discrepancy in the PK of voriconazole could be explained by blood sampling time data, a difference in the proportion of sample characteristics, and co-administration with *CYP2C19* inhibitors. In our study, most patients received PPIs (omeprazole 20 mg/day 44.62%, omeprazole ≥ 40 mg/day 26.15%, esomprazole 3.08%, and rabeprazole 1.54%). In contrast, in the Mangal et al. study, most patients received pantoprazole 70.6% [17]. Han et al. studied the population PK of lung transplant recipients, and collected full intensive PK data from the second dose injection of an initial dose 6 mg/kg of intravenous (IV) form, and 5th–35th dose 200 mg of oral form voriconazole [22]. Dalton et al. [13] studied 3352 blood samples from 63 healthy volunteers and 177 patients that were collected 1–29 times/dose of single or multiple dose, that varied in dose IV or oral form. Liu et al. [15] studied subjects (305 subjects, 965 blood samples) whose collected samples tended to have a peak concentration, middle concentration (time between peak and trough), and trough concentration of voriconazole on day 3, day 7, and day 14, post dose, with dosing of voriconazole of 6 mg/kg IV q 12 h 2 doses, then 4 mg/kg IV q 12 h, switching to 300 mg (150 mg for body weigh <40 kg) for oral form q 12 h when patients could feed/eat. Friberg et al. [23] studied both pediatric and adult patients, which was different from our study.

Covariate analysis revealed that albumin level and omeprazole ≥ 40 mg/day have a significant impact on CL/F, and therefore they were included in the final model. The PK parameters were generally well estimated in the final model. The estimations had acceptable precision, and the goodness-of-fit plots indicated a reasonable fit to the data. The results of bootstrap analysis showed the robustness of the model, and prediction-corrected visual predictive checks showed the model to be adequate for describing the data.

Since there were a limited number of samples drawn during the absorption phase, we fixed the Ka at 1.1 h^−1^ [25]. Interpatient variability in the V/F could not be estimated due to inadequate blood samples during the distribution phase. The estimation of CL/F was 3.43 L/h, which was within the range of previously reported values in population analysis (range from 2.88 to 11.2 L/h) [14,16,18,19,21,22,25]. The estimation of V/F was 47.6 L, which was lower than the values observed in other populations. However, the value of V/F was within the range reported (22.47–248 L) in previous studies [14,16,18,19,21,25,31,32]. In addition, our study could not identify body weight as a covariate since we performed allometric scaling.

Plasma albumin was found to be a factor that significantly influenced the CL/F of voriconazole. A study by Vanstraelen et al. found a positive relationship between voriconazole plasma protein binding and plasma albumin concentration, which could be due to a higher unbound voriconazole concentration with decreasing albumin concentration [33]. Nevertheless, an explanation of low plasma albumin level associated with the reduction of CL/F of voriconazole was still inconclusive. In our opinion, an increase in voriconazole metabolism is related to an unbound form. However, voriconazole is a low extraction ratio (0.09–0.39) [34], the saturated enzyme for drug metabolism limited the clearance of voriconazole. Moreover, since almost all of the patients in our study took the same dose of voriconazole, the Michaelis–Menten equation could not be clearly demonstrated. Dote et al. [29] and Hirata et al. [35] found hypoalbuminemia to be associated with decreasing clearance of voriconazole, therefore, toxic vigilance is important in patients with low albumin.

Concurrent administration of PPIs is common in patients receiving voriconazole. A physiologically based PK study showed that the impact of PPIs on the PK of voriconazole was related to the dose and inhibitory effect of *CYP2C19*. The area under the curve (AUC) of voriconazole was increased by 39%, 18%, 12%, and 1% with co-administration of omeprazole 40 mg/day, esomeprazole 40 mg/day, lansoprazole 30 mg/day, and rabeprazole 20 mg/day, respectively [36]. A study by Wood et al. Investigated the effect of omeprazole 40 mg/day on the steady-state PK of voriconazole in 18 healthy male volunteers. Their study found that C_max_ and AUC were increased by 15% and 41%, respectively [37]. The mechanism behind the interaction with CYP450 inhibitors, omeprazole, and voriconazole is via CYP2C19 and CYP3A4 inhibitors [4,38,39]. On the one hand, the effect of PPIs on the PK of voriconazole depends on the dose and kind of PPIs, and how these factors affect various capabilities of *CYP2C19* [12,36]. On the other hand, a recommendation specific to omeprazole 20 mg/day stated that this drug and dosage did not significantly affect voriconazole levels, which was similar to our study [4,40].

Conflicting findings relative to the impact of *CYP2C19* polymorphisms on voriconazole clearance have been observed among some population PK analyses. Previous studies have found the poor metabolizer group (11.3–19%) [16,18,19] to be adversely affected relative to the clearance of voriconazole. In our study, the data showed a poor metabolizer group of 10.77% (CYP2C19*2/*2 7.69%, and CYP2C19*2/*3 3.08%), which indicates *CYP2C19* polymorphisms in the analysis, but we were not able to find any influence of gene polymorphism. This observed absence of association is likely due to the modest effect on clearance of voriconazole among extensive, intermediate, and poor metabolizer, in each group. Furthermore, it is possible that the effect of *CYP2C19* expressers could be minimized by omeprazole ≥ 40 mg/day and plasma albumin effects, which could mask the effect of gene polymorphisms.

Final model-based simulations were performed to compare each dosage regimen with the percentages of C_min, ss_ of voriconazole that achieved therapeutic range (1–5 mg/L) [2,3,9,10]. Several dosage regimens of a maintenance dose of voriconazole after loading (50, 100, 150, 200, 250, 300, 350, and 400 mg orally every 12 h) were used to predict C_min, ss_ under different covariate combinations and conditions. This is the first study to establish the influence of plasma albumin level and co-administration of omeprazole and voriconazole on dose-adjusted concentration of voriconazole in patients with hematologic diseases. The results of dosing simulations are presented in Figure 3 and Figure 4. In the non-omeprazole or omeprazole 20 mg/day group (Figure 3), patients with albumin 1.5–3, 3.01–4, and 4.01–4.5 g/dL that received a dose of voriconazole 50, 100, and 200 mg orally every 12 h, respectively, had the highest proportion of patients that reached the therapeutic concentration (C_min, ss_ 1–5 mg/L) at steady state. Among patients who received omeprazole ≥ 40 mg/day (Figure 4), those with albumin 1.5–3 g/dL who received a dose of voriconazole 50 mg orally every 12 h had the highest percentage of patients who reached the therapeutic target. In contrast, patients with albumin 3.01–4.5 g/dL who received omeprazole ≥ 40 mg/day and a dose of voriconazole 100 mg orally every 12 h had the highest number of patients that achieved a therapeutic range. Moreover, the optimal regimens of voriconazole should lower the percentages of patients that achieve toxic or sub-therapeutic range. The application of our simulation (Figure 3 and Figure 4) for predicting C_min, ss_ was found to be appropriate for a plasma albumin range of about 1.6–4.5 g/dL.

Some limitations of this study should be noted. Firstly, the time of blood collection varied from 0.75–12 h. However, most of the blood samples that were collected in this study had a trough level that was consistent with the predicted trough concentration of voriconazole. Secondly, data specific to the absorption phase (0–2 h) and distribution phase were limited. Thus, Ka and V/F and their variability could not be well estimated. Finally, the effect of simulation dose on clinical outcome and toxicity was not evaluated in this study. Further studies should be conducted to investigate this effect.

## 4. Materials and Methods

### 4.1. Patients and Ethics

A PK study of oral voriconazole was conducted in Thai adult patients with hematologic diseases at Siriraj Hospital, which is a 2300 bed university-based national super tertiary referral center that is located in Bangkok, Thailand.

Patients with hematologic diseases were treated with voriconazole for prevention or treatment of invasive aspergillosis following the guidelines of the European Organization for Research and Treatment of Cancer/Invasive Fungal Infections Cooperative Group: EORTC and the National Institute of Allergy and Infectious Diseases Mycoses Study Group: MSG or EORTC/MSG 2008 [41]. The inclusion criteria were Thai adult patients aged ≥ 18 years with hematologic diseases. Voriconazole was administered orally according to patient’s body weight. The loading dose was 400 mg every 12 h (2 doses in all 65 patients) on the first day, followed by a maintenance dose of 200 mg every 12 h for weight ≥ 40 kg. In patients with weight < 40 kg, the maintenance dose depended on doctor’s discretion (see the information in Supplementary Materials). Blood samples were collected in 18 subjects on the seventh day of voriconazole (7 blood samples; 0 (pre-dose), 1, 1.5, 2, 4, 8, and 12 h) during August 2016 to October 2018, and a trough blood level at different times was collected in another group of 47 subjects from the 7th to the 40th day of maintenance dose of voriconazole. Patients who were pregnant, unable to take or tolerate voriconazole until the end of the specified date, had severe hepatic disease (Child Pugh C), or had voriconazole hypersensitivity were excluded.

The protocol for this study was approved by the Siriraj Institutional Review Board (SIRB) of the Faculty of Medicine Siriraj Hospital, Mahidol University, Bangkok, Thailand (COA numbers 610/2558 (EC1) and 104/2554 (EC2)). Written informed consent was obtained from all patients.

### 4.2. Measurement of Voriconazole Plasma Concentrations

Blood samples were collected in an ethylene diamine tetra-acetic acid (EDTA) tube and kept at −80 °C until drug concentration analysis. Plasma voriconazole concentrations were measured by validated method with high-performance liquid chromatography (HPLC) assay, as previously described [42]. All samples were analyzed by the Infectious Laboratory, Department of Preventive and Social Medicine, Faculty of Medicine Siriraj Hospital, Mahidol University. The lower limit of quantification was 0.2 mg/L (the linearity ranged from 0.2 to 20 mg/L). The correlation coefficient value was 0.98. The CV% of voriconazole concentrations had an intra-day variability range of 0.78–3.01%, and an inter-day variability of 1.52–4%. The ranges for accuracy and extraction recovery were 99.3–101% and 99.2–101%, respectively [42].

### 4.3. Data Collection

Patient data that were collected from medical records included the following: demographic data (i.e., age, gender, comorbidity, and body weight); source of fungal infection; drugs taken concurrently with voriconazole, such as PPIs, sulfamethoxazole/trimethoprim, hormones, and steroids; and laboratory data, including white blood cell, absolute neutrophil count, hemoglobin, hematocrit, platelets, blood urea nitrogen, serum creatinine, AST, ALT, ALP, DB, TB, albumin, globulin, gene polymorphism of *CYP2C19*, and plasma voriconazole concentrations.

### 4.4. CYP2C19 Genotype Analysis

DNA was extracted from a buccal swab using a Gentra Puregene Blood Kit (QIAGEN, Hilden, Germany). *CYP2C19* polymorphisms were genotyped by restriction fragment length polymorphism (RFLP). *CYP2C19* *2 (c.681G > A), *3 (c.636G > A), and *17 (c. −806C > T) were digested with *Msp*I, *Bam*HI, and *SfaN*I, respectively. The phenotype of *CYP2C19* was classified into 4 groups [43], as follows: (1) ultra-rapid metabolizer (UM) CYP2C19*1/*17 and CYP2C19*17/*17; (2) extensive metabolizer (EM) CYP2C19*1/*1; (3) intermediate metabolizer (IM) CYP2C19*1/*2, *1/*3, *2/*17, *3/*17; and, (4) poor metabolizer (PM) CYP2C19*2/*2, *2/*3, *3/*3.

### 4.5. Population Pharmacokinetic Modeling Method

A population PK model was established using nonlinear mixed-effects modeling. The PK parameters and their variabilities were estimated using first-order conditional estimation with interaction (FOCE-I) method. The software packages used for modeling included NONMEM (version VII; Icon Development Solutions, Ellicott City, MD, USA), Perl-speaks-NONMEM (PsN) Toolkit (version 3.7.6: http://psn.sourseforge.net/), and Pirana (version 2.8.1.). The graphical analysis was performed using R package (version 3.1.2, R Development Core team; http://www.r-project.org) and Xpose program (version 4.5.0).

The PK characteristics of oral voriconazole were investigated for fit to the data using one- and two-compartment model with first-order absorption with and without absorption lag time, and linear or nonlinear (Michaelis–Menten) elimination.

The inter-individual variability (IIV) was described with an exponential model; thus, a log-normal distribution of the PK parameter was assumed: CL/F = θ × (EXP) η_i_, where CL/F is the individual apparent oral clearance (CL/F) of voriconazole, θ is the typical value in the population CL/F, and η_i_ is the IIV which is assumed to be normally distributed with a mean of zero and variance of ω^2^.

The residual variability was tested with the following models:

Additive model (C_obs,ij_ = C_pred,ij_ + ε_ij_);
Proportional model (C_obs,ij_ = C_pred,ij_ × (1 + ε_ij_));
Combined proportional with additive model (C_obs,ij_ = C_pred,ij_ × (1 + ε1_ij_) + ε2_ij_).

where C_obs,ij_ is the ith observed concentration of the jth individual; C_pred,ij_ is the ith predicted concentration of the jth individual; ε_ij_ is the RUV, which was assumed to be normally distributed with a mean of zero and a variance of σ^2^; ε1 is the RUV with proportional function; and ε2 is the RUV with additive function.

The influence of body weight on apparent clearance (CL/F) and the volume of distribution (V/F) were explored using allometric scaling as follows: CL/F = θ_1_ × (WT_i_/WT_std_) ^0^^.75^ and V/F = θ_2_ × (WT_i_/WT_std_) ^1^, where θ_1_ and θ_2_ are the mean parameters to be estimated for CL/F and V/F, respectively; WT_i_ is the individual body weight (kg); and WT_std_ is the standard body weight, which is the median weight of the population.

The influence of clinical and genetic factors on PK parameters was explored graphically to identify potential relationships, and then covariate analysis was performed using a stepwise approach. The difference in OFV > 3.84 (χ^2^, df = 1, *p* < 0.05) and 6.63 (χ^2^, df = 1, *p* < 0.01) was used as cut-off criteria for forward inclusion and backward deletion, respectively.

The effect of covariates on PK parameters was evaluated using different functions based on the type of covariate. Continuous covariates (e.g., age, body weight, albumin level, AST, ALT, ALP, TB, and DB) were centered by their median and were explored with linear, power, and exponential model. Categorical covariates (e.g., gender, *CYP2C19* genotypes or phenotypes, dosing of omeprazole (20 mg/day and ≥40 mg/day), sulfamethoxazole/trimethoprim, and hormones) were explored with additive, fractional, and exponential model.

### 4.6. Model Evaluation and Validation

Model selection was guided by the difference in objective function value (OFV), improvement in the goodness-of-fit (GOF) plots, and the precision of parameter estimates (%RSE). Model evaluation was performed using prediction-corrected visual predictive checks (pcVPC) and bootstrap method (*n* = 1000 samples with replacement from the original dataset).

### 4.7. Simulation

Monte Carlo simulation was performed to determine optimal dosing regimens of voriconazole. A simulation of minimal voriconazole concentrations (C_min, ss_) at steady state was conducted using the parameter estimates from the final model. A total of 2000 replicates of C_min, ss_ were simulated for each dosage regimen, and the influence of each significant covariate was evaluated. A C_min, ss_ of voriconazole between 1 and 5 mg/L represents the therapeutic target of drug efficacy [2,3,9,10].

### 4.8. Statistical Analysis

Patient characteristics were analyzed by STATA software (version 14.2) (StataCorp, LLC, College Station, TX, USA). Categorical data are reported as frequency and percentage, and continuous data are summarized as mean ± standard deviation (SD) and range or median and interquartile range (IQR).

## 5. Conclusions

The population PK model of voriconazole has been developed, and its PK profile has been characterized in Thai adult patients with hematologic diseases. The final model indicated that plasma albumin level and omeprazole ≥ 40 mg/day significantly influenced the clearance of voriconazole. The simulations suggested a decreased dose of voriconazole in patients who receive omeprazole ≥ 40 mg/day. In addition, patients with a lower albumin level should receive a lower dose of voriconazole than the dose that would be given to patients with a normal albumin level. Voriconazole should be used with caution in hypoalbuminemia patients who receive omeprazole ≥ 40 mg/day. Thus, therapeutic drug monitoring can aid dosage adjustment in patients who are treated with voriconazole. The results of this study could be applied to design a dosing optimization model of voriconazole that included significant covariates identified in Thai population.

## Figures and Tables

**Table 1:** Baseline demographic and clinical characteristics of study patients (*n* = 65).

Characteristics	Values
Gender, *n* (%)	Male: 41 (63.08), Female: 24 (36.92)
Age *, yrs, mean ± SD (range)	47.65 ± 15.22 (20–78)
Body weight *, kg, mean ± SD (range)	58.57 ± 13.83 (27.20–105.00)
<40 kg, *n* (%)	4 (6.15)
40–90 kg, *n* (%)	59 (90.77)
>90 kg, *n* (%)	2 (3.08)
Underlying disease, *n* (%)	
Hematologic diseases	AML 37 ** (56.92), Lymphoma 14 (21.54), ALL 9 (13.85), Others *** 5 (7.69)
Hypertension	3 (4.62)
Coronary artery disease	2 (3.08)
Liver disease	2 (3.08)
Others ****	6 (9.23)
Baseline laboratory test, median (IQR)	
Serum creatinine, mg/dL	1.00 (0.70–1.3)
Aspartate aminotransferase, U/L	30.00 (22–49)
Alanine aminotransferase, U/L	26.00 (15–40)
Alkaline phosphatase, U/L	151.00 (105–216)
Total bilirubin, mg/dL	0.61 (0.40–1.00)
Direct bilirubin, mg/dL	0.35 (0.23–0.73)
White blood cell, cells/mm^3^	550 (270–2410)
Absolute neutrophil count, cells/mm^3^	120.00 (10–1350)
Platelets, ×10^3^/mm^3^	28.00 (12.00–54.00)
Baseline laboratory test, mean ± SD (range)	
Albumin *, g/dL	3.04 ± 0.61 (1.60–4.3)
Globulin *, g/dL	3.47 ± 0.96 (1.50–6.5)
Hemoglobin *, g/dL	8.45 ± 1.25 (5.50–12.00)
Hematocrit *, %	24.36 ± 3.99 (14.90–38.10)
*CYP2C19* polymorphism, *n* (%)	
Ultra-rapid metabolizer (UM) (* 1/* 17)	1 (1.54)
Extensive metabolizer (EM) (* 1/* 1)	33 (50.77)
Intermediate metabolizer (IM) (* 1/* 2, * 1/* 3, * 2/* 17)	24 (36.92)
Poor metabolizer (PM) (* 2/* 2, * 2/* 3)	7 (10.77)
Voriconazole dosing * (mg/kg/day), mean ± SD (range)	7.18 ± 1.76 (3.81–14.71)
Current medication, *n* (%)	
Omeprazole 20 mg/day	29 (44.62)
Omeprazole ≥ 40 mg/day	17 (26.15)
Esomeprazole	2 (3.08)
Rabeprazole	1 (1.54)
Sulfamethoxazole/trimethoprim	19 (29.23)
Hormones	6 (9.23)
Steroids	3 (4.62)

* Use mean ± SD (range) because the type of data was a normal distribution. *** Myelodysplastic syndrome, 2 patients (from 37 ** AML patients); chronic myeloid leukemia, 2 patients; aplastic anemia, 2 patients; and prolymphocytic leukemia, 1 patient. **** Human immunodeficiency virus, 1 patient; benign prostatic hyperplasia, 1 patient; psoriasis, 1 patient; glaucoma, 1 patient; breast cancer, 1 patient; and prostate cancer, 1 patient. AML, acute myeloid leukemia; ALL, acute lymphoblastic leukemia; SD, standard deviation; IQR, interquartile range.

**Table 2:** Population pharmacokinetic parameters of voriconazole.

Parameters	NONMEM Results				Bootstrap Results (100% Successful)		
	Estimated Value	SE	2.5th *	97.5th *	Median	2.5th	97.5th
CL/F (L/h)	3.430	0.287	2.867	3.993	3.430	2.919	4.095
V/F (L)	47.600	6.600	34.664	60.536	47.811	35.621	63.973
Ka (/h)	FIX 1.100						
CL-albumin	0.249	0.0925	0.068	0.430	0.250	0.057	0.413
CL-omeprazole ≥ 40 mg/day	−0.306	0.084	−0.471	−0.141	−0.300	−0.458	−0.058
IIV-CL	0.226	0.0524	0.123	0.329	0.216	0.124	0.339
(%CV)	50.40%		36.18%	62.42%	49.10%	36.33%	63.53%
RUV (mg/L)	2.67	0.706	1.286	4.054	2.576	1.546	4.373

* Calculated from the estimated value ± 1.96 × SE. NONMEM, nonlinear mixed effects modeling; CL/F, apparent oral clearance; V/F, apparent volume of distribution; Ka, absorption rate constant; CL, clearance; IIV, interindividual variability; %CV, percent coefficient variation; RUV, residual variability; SE, standard error.

## Supplementary Materials

The following are available online at https://www.mdpi.com/2079-6382/9/9/574/s1, Figure S1: A visual predictive checks (VPC) plot of the final model. Open circles show observed plasma concentrations, and the solid line and dashed line show the median and 95% confidence interval (CI) of observations, respectively. The red shaded area and the blue shaded area show the 95% CI of the median and the 5th and 95th percentiles of the consequence of 1000 simulations of the final model, respectively, S1: Code of the one-compartment model, S2: Patients with body weight <40 kg, Table S1: The sensitivity analysis results by base model showed that the clearance estimates were consistent across different values of the absorption rate constant (Ka) 0.163–1.38.

## References

[1] Segal B.H. (2009). Aspergillosis. N. Engl. J. Med..

[2] Patterson T.F., Thompson G.R., Denning D.W., Fishman J.A., Hadley S., Herbrecht R., Kontoyiannis D.P., Marr K.A., Morrison V.A., Nguyen M.H. (2016). Practice guidelines for the diagnosis and management of aspergillosis: 2016 update by the Infectious Diseases Society of America. Clin. Infect. Dis..

[3] Ullmann A.J., Aguado J.M., Arikan-Akdagli S., Denning D.W., Groll A.H., Lagrou K., Lass-Florl C., Lewis R.E., Munoz P., Verweij P.E. (2018). Diagnosis and management of Aspergillus diseases: Executive summary of the 2017 ESCMID-ECMM-ERS guideline. Clin. Microbiol. Infect..

[4] Pfizer Vfend^®^, Voriconazole. https://www.accessdata.fda.gov/drugsatfda_docs/label/2019/021266s039,021267s050,021630s029lbl.pdf.

[5] Herbrecht R., Denning D.W., Patterson T.F., Bennett J.E., Greene R.E., Oestmann J.W., Kern W.V., Marr K.A., Ribaud P., Lortholary O. (2002). Voriconazole versus amphotericin B for primary therapy of invasive aspergillosis. N. Engl. J. Med..

[6] Tissot F., Agrawal S., Pagano L., Petrikkos G., Groll A.H., Skiada A., Lass-Florl C., Calandra T., Viscoli C., Herbrecht R. (2017). ECIL-6 guidelines for the treatment of invasive candidiasis, aspergillosis and mucormycosis in leukemia and hematopoietic stem cell transplant patients. Haematologica.

[7] Wingard J.R., Carter S.L., Walsh T.J., Kurtzberg J., Small T.N., Baden L.R., Gersten I.D., Mendizabal A.M., Leather H.L., Confer D.L. (2010). Randomized, double-blind trial of fluconazole versus voriconazole for prevention of invasive fungal infection after allogeneic hematopoietic cell transplantation. Blood.

[8] Marks D.I., Pagliuca A., Kibbler C.C., Glasmacher A., Heussel C.P., Kantecki M., Miller P.J., Ribaud P., Schlamm H.T., Solano C. (2011). Voriconazole versus itraconazole for antifungal prophylaxis following allogeneic haematopoietic stem-cell transplantation. Br. J. Haematol..

[9] Hamada Y., Tokimatsu I., Mikamo H., Kimura M., Seki M., Takakura S., Ohmagari N., Takahashi Y., Kasahara K., Matsumoto K. (2013). Practice guidelines for therapeutic drug monitoring of voriconazole: A consensus review of the Japanese Society of Chemotherapy and the Japanese Society of Therapeutic Drug Monitoring. J. Infect. Chemother. Off. J. Jpn. Soc. Chemother..

[10] Ashbee H.R., Barnes R.A., Johnson E.M., Richardson M.D., Gorton R., Hope W.W. (2014). Therapeutic drug monitoring (TDM) of antifungal agents: Guidelines from the British Society for Medical Mycology. J. Antimicrob. Chemother..

[11] Dolton M.J., McLachlan A.J. (2014). Voriconazole pharmacokinetics and exposure-response relationships: Assessing the links between exposure, efficacy and toxicity. Int. J. Antimicrob. Agents.

[12] Shi C., Xiao Y., Mao Y., Wu J., Lin N. (2019). Voriconazole: A review of population pharmacokinetic analyses. Clin. Pharmacokinet..

[13] Dolton M.J., Mikus G., Weiss J., Ray J.E., McLachlan A.J. (2014). Understanding variability with voriconazole using a population pharmacokinetic approach: Implications for optimal dosing. J. Antimicrob. Chemother..

[14] Li Z.W., Peng F.H., Yan M., Liang W., Liu X.L., Wu Y.Q., Lin X.B., Tan S.L., Wang F., Xu P. (2017). Impact of CYP2C19 Genotype and Liver Function on Voriconazole Pharmacokinetics in Renal Transplant Recipients. Ther. Drug Monit..

[15] Liu P., Mould D.R. (2014). Population pharmacokinetic analysis of voriconazole and anidulafungin in adult patients with invasive aspergillosis. Antimicrob. Agents Chemother..

[16] Lin X.B., Li Z.W., Yan M., Zhang B.K., Liang W., Wang F., Xu P., Xiang D.X., Xie X.B., Yu S.J. (2018). Population pharmacokinetics of voriconazole and CYP2C19 polymorphisms for optimizing dosing regimens in renal transplant recipients. Br. J. Clin. Pharmacol..

[17] Mangal N., Hamadeh I.S., Arwood M.J., Cavallari L.H., Samant T.S., Klinker K.P., Bulitta J., Schmidt S. (2018). Optimization of voriconazole therapy for the treatment of invasive fungal infections in adults. Clin. Pharmacol. Ther..

[18] Wang T., Chen S., Sun J., Cai J., Cheng X., Dong H., Wang X., Xing J., Dong W., Yao H. (2014). Identification of factors influencing the pharmacokinetics of voriconazole and the optimization of dosage regimens based on Monte Carlo simulation in patients with invasive fungal infections. J. Antimicrob. Chemother..

[19] Liu Y., Qiu T., Liu Y., Wang J., Hu K., Bao F., Zhang C. (2019). Model-based voriconazole dose optimization in Chinese adult patients with hematologic malignancies. Clin. Ther..

[20] Kim Y., Rhee S.J., Park W.B., Yu K.S., Jang I.J., Lee S. (2019). A Personalized CYP2C19 phenotype-guided dosing regimen of voriconazole using a population pharmacokinetic analysis. J. Clin. Med..

[21] Chen C., Yang T., Li X., Ma L., Liu Y., Zhou Y., Ren H., Cui Y. (2019). Population pharmacokinetics of voriconazole in Chinese patients with hematopoietic stem cell transplantation. Eur. J. Drug Metab. Pharmacokinet..

[22] Han K., Capitano B., Bies R., Potoski B.A., Husain S., Gilbert S., Paterson D.L., McCurry K., Venkataramanan R. (2010). Bioavailability and population pharmacokinetics of voriconazole in lung transplant recipients. Antimicrob. Agents Chemother..

[23] Friberg L.E., Ravva P., Karlsson M.O., Liu P. (2012). Integrated population pharmacokinetic analysis of voriconazole in children, adolescents, and adults. Antimicrob. Agents Chemother..

[24] Chen W., Xie H., Liang F., Meng D., Rui J., Yin X., Zhang T., Xiao X., Cai S., Liu X. (2015). Population pharmacokinetics in China: The dynamics of intravenous voriconazole in critically ill patients with pulmonary disease. Biol. Pharm. Bull..

[25] Pascual A., Csajka C., Buclin T., Bolay S., Bille J., Calandra T., Marchetti O. (2012). Challenging recommended oral and intravenous voriconazole doses for improved efficacy and safety: Population pharmacokinetics-based analysis of adult patients with invasive fungal infections. Clin. Infect. Dis..

[26] Leitinger E., Hui L., Grigg A. (2019). Is there a role for proton pump inhibitor prophylaxis in haematology patients?. Intern. Med. J..

[27] Chayakulkeeree M., Poovipirom N., Siengwattana P., Maneerattanaporn M. (2015). Effect of proton pump inhibitor on plasma voriconazole concentration in Thai patients. J. Med. Assoc. Thai..

[28] Deluche E., Girault S., Jesus P., Monzat S., Turlure P., Leobon S., Abraham J., Daly N., Dauriac O., Bordessoule D. (2017). Assessment of the nutritional status of adult patients with acute myeloid leukemia during induction chemotherapy. Nutrition.

[29] Dote S., Sawai M., Nozaki A., Naruhashi K., Kobayashi Y., Nakanishi H. (2016). A retrospective analysis of patient-specific factors on voriconazole clearance. J. Pharm. Health Care Sci..

[30] Sukasem C., Tunthong R., Chamnanphon M., Santon S., Jantararoungtong T., Koomdee N., Prommas S., Puangpetch A., Vathesatogkit P. (2013). CYP2C19 polymorphisms in the Thai population and the clinical response to clopidogrel in patients with atherothrombotic-risk factors. Pharmgen. Pers. Med..

[31] Nomura K., Fujimoto Y., Kanbayashi Y., Ikawa K., Taniwaki M. (2008). Pharmacokinetic-pharmacodynamic analysis of voriconazole in Japanese patients with hematological malignancies. Eur. J. Clin. Microbiol. Infect. Dis..

[32] Han K., Bies R., Johnson H., Capitano B., Venkataramanan R. (2011). Population pharmacokinetic evaluation with external validation and Bayesian estimator of voriconazole in liver transplant recipients. Clin. Pharmacokinet..

[33] Vanstraelen K., Wauters J., Vercammen I., de Loor H., Maertens J., Lagrou K., Annaert P., Spriet I. (2014). Impact of hypoalbuminemia on voriconazole pharmacokinetics in critically ill adult patients. Antimicrob. Agents Chemother..

[34] Johnson H.J., Han K., Capitano B., Blisard D., Husain S., Linden P.K., Marcos A., Kwak E.J., Potoski B., Paterson D.L. (2010). Voriconazole pharmacokinetics in liver transplant recipients. Antimicrob. Agents Chemother..

[35] Hirata A., Noto K., Ota R., Yokoyama S., Hosomi K., Takada M., Matsuoka H. (2019). Voriconazole trough concentration and hepatotoxicity in patients with low serum albumin. Int. J. Clin. Pharmacol. Ther..

[36] Qi F., Zhu L., Li N., Ge T., Xu G., Liao S. (2017). Influence of different proton pump inhibitors on the pharmacokinetics of voriconazole. Int. J. Antimicrob. Agents.

[37] Wood N., Tan K., Purkins L., Layton G., Hamlin J., Kleinermans D., Nichols D. (2003). Effect of omeprazole on the steady-state pharmacokinetics of voriconazole. Br. J. Clin. Pharmacol..

[38] Birkett D.J., Andersson T., Miners J.O. (1996). Assays of omeprazole metabolism as a substrate probe for human CYP isoforms. Methods Enzymol..

[39] Andersson T., Miners J.O., Veronese M.E., Birkett D.J. (1994). Identification of human liver cytochrome P450 isoforms mediating secondary omeprazole metabolism. Br. J. Clin. Pharmacol..

[40] Chau M.M., Kong D.C., van Hal S.J., Urbancic K., Trubiano J.A., Cassumbhoy M., Wilkes J., Cooper C.M., Roberts J.A., Marriott D.J. (2014). Consensus guidelines for optimising antifungal drug delivery and monitoring to avoid toxicity and improve outcomes in patients with haematological malignancy, 2014. Intern. Med. J..

[41] De Pauw B., Walsh T.J., Donnelly J.P., Stevens D.A., Edwards J.E., Calandra T., Pappas P.G., Maertens J., Lortholary O., Kauffman C.A. (2008). Revised definitions of invasive fungal disease from the European Organization for Research and Treatment of Cancer/Invasive Fungal Infections Cooperative Group and the National Institute of Allergy and Infectious Diseases Mycoses Study Group (EORTC/MSG) Consensus Group. Clin. Infect. Dis..

[42] Sangsiriwut K., Chayakulkeeree M. (2013). Rapid high performance liquid chromatographic assay for determination of voriconazole concentration in human plasma. J. Med. Assoc. Thai..

[43] Moriyama B., Obeng A.O., Barbarino J., Penzak S.R., Henning S.A., Scott S.A., Agundez J., Wingard J.R., McLeod H.L., Klein T.E. (2017). Clinical pharmacogenetics implementation consortium (CPIC) guidelines for CYP2C19 and voriconazole therapy. Clin. Pharmacol. Ther..

