# Supplementary material for: Impact of Albumin and Omeprazole on Steady-State Population Pharmacokinetics of Voriconazole and Development of a Voriconazole Dosing Optimization Model in Thai Patients with Hematologic Diseases
Source: Antibiotics (Basel). 2020 Sep 3;9(9):574. doi: 10.3390/antibiotics9090574 (PMC7557832; doi:10.3390/antibiotics9090574)
Supplement: Supplementary file 1 [file antibiotics-09-00574-s001.pdf]

Supplementary Materials

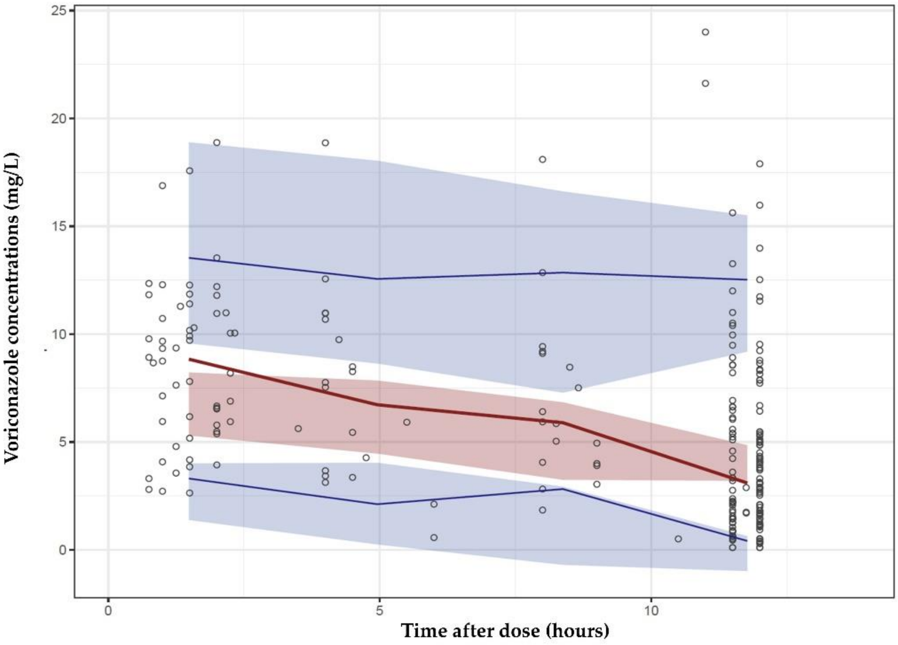

Figure S1. A visual predictive checks (VPC) plot of the final model. Open circles show observed plasma concentrations, and the solid line and dashed line show the median and 95% confidence interval (CI) of observations, respectively. The red shaded area and the blue shaded area show the 95% CI of the median and the 5th and 95th percentiles of the consequence of 1,000 simulations of the final model, respectively.

S1. Code of the one-compartment model

```
$SUBROUTINE ADVAN2 TRANS2

$PK
  TVCL=THETA(1)
  CL = TVCL*EXP(ETA(1))

  TVV=THETA(2)
  V=TVV*EXP(ETA(2))

  TVKA = THETA(3)
  KA = TVKA*EXP(ETA(3))
```

S2. Patients with body weight <40 kg

Of the 65 included patients, 4 patients had a body weight <40 kg. These patients took a loading dose of voriconazole 400 mg orally every 12 h (2 doses), which was followed by a maintenance dose of 200 mg orally every 12 h in 3 patients, and 1 patient took 200 mg at 7.00 a.m. and 100 mg at 7.00 p.m.

Table S1. The sensitivity analysis results by base model showed that the clearance estimates were consistent across different values of the absorption rate constant (Ka) 0.163-1.38.

Studies	The absorption rate constant (Ka), /h	Clearance (CL/F), L/h
Nomura, et al. [1]	0.163	2.99
Dolton, et al. [2]	0.53	3.03
Han, et al. [3]	0.591	3.04
Mangal, et al. [4]	0.654	3.05
Liu, et al. [5]	0.729	3.06
Karlsson, et al. [6]	0.849	3.08
Pascual, et al., Wang, et al., Li, et al., Lin, et al., and Chen, et al. [7-11]	1.1	3.12
Gastine, et al. [12]	1.19	3.13
Liu, et al. [13]	1.2	3.13
Muto, et al. [14]	1.38	3.15

References

1. Nomura, K.; Fujimoto, Y.; Kanbayashi, Y.; Ikawa, K.; Taniwaki, M. Pharmacokinetic-pharmacodynamic analysis of voriconazole in Japanese patients with hematological malignancies. *Eur. J. Clin. Microbiol. Infect. Dis.* **2008**, *27*, 1141-1143, doi:10.1007/s10096-008-0543-1.
2. Dolton, M.J.; Mikus, G.; Weiss, J.; Ray, J.E.; McLachlan, A.J. Understanding variability with voriconazole using a population pharmacokinetic approach: implications for optimal dosing. *J. Antimicrob. Chemother.* **2014**, *69*, 1633-1641, doi:10.1093/jac/dku031.
3. Han, K.; Capitano, B.; Bies, R.; Potoski, B.A.; Husain, S.; Gilbert, S.; Paterson, D.L.; McCurry, K.; Venkataramanan, R. Bioavailability and population pharmacokinetics of voriconazole in lung transplant recipients. *Antimicrob. Agents Chemother.* **2010**, *54*, 4424-4431, doi:10.1128/aac.00504-10.
4. Mangal, N.; Hamadeh, I.S.; Arwood, M.J.; Cavallari, L.H.; Samant, T.S.; Klinker, K.P.; Bulitta, J.; Schmidt, S. Optimization of Voriconazole Therapy for the Treatment of Invasive Fungal Infections in Adults. *Clin. Pharmacol. Ther.* **2018**, *104*, 957-965, doi:10.1002/cpt.1012.
5. Liu, Y.; Qiu, T.; Liu, Y.; Wang, J.; Hu, K.; Bao, F.; Zhang, C. Model-based Voriconazole Dose Optimization in Chinese Adult Patients With Hematologic Malignancies. *Clin. Ther.* **2019**, *41*, 1151-1163, doi:10.1016/j.clinthera.2019.04.027.
6. Karlsson, M.O.; Lutsar, I.; Milligan, P.A. Population pharmacokinetic analysis of voriconazole plasma concentration data from pediatric studies. *Antimicrob. Agents Chemother.* **2009**, *53*, 935-944, doi:10.1128/aac.00751-08.
7. Pascual, A.; Csajka, C.; Buclin, T.; Bolay, S.; Bille, J.; Calandra, T.; Marchetti, O. Challenging recommended oral and intravenous voriconazole doses for improved efficacy and safety: population pharmacokinetics-based analysis of adult patients with invasive fungal infections. *Clin. Infect. Dis.* **2012**, *55*, 381-390, doi:10.1093/cid/cis437.
8. Wang, T.; Chen, S.; Sun, J.; Cai, J.; Cheng, X.; Dong, H.; Wang, X.; Xing, J.; Dong, W.; Yao, H., et al. Identification of factors influencing the pharmacokinetics of voriconazole and the optimization of dosage regimens based on Monte Carlo simulation in patients with invasive fungal infections. *J. Antimicrob. Chemother.* **2014**, *69*, 463-470, doi:10.1093/jac/dkt369.
9. Li, Z.W.; Peng, F.H.; Yan, M.; Liang, W.; Liu, X.L.; Wu, Y.Q.; Lin, X.B.; Tan, S.L.; Wang, F.; Xu, P., et al. Impact of CYP2C19 Genotype and Liver Function on Voriconazole Pharmacokinetics in

Renal Transplant Recipients. *Ther. Drug Monit.* **2017**, *39*, 422-428, doi:10.1097/ftd.0000000000000425.
10. Lin, X.B.; Li, Z.W.; Yan, M.; Zhang, B.K.; Liang, W.; Wang, F.; Xu, P.; Xiang, D.X.; Xie, X.B.; Yu, S.J., et al. Population pharmacokinetics of voriconazole and CYP2C19 polymorphisms for optimizing dosing regimens in renal transplant recipients. *Br. J. Clin. Pharmacol.* **2018**, *84*, 1587-1597, doi:10.1111/bcp.13595.
11. Chen, C.; Yang, T.; Li, X.; Ma, L.; Liu, Y.; Zhou, Y.; Ren, H.; Cui, Y. Population Pharmacokinetics of Voriconazole in Chinese Patients with Hematopoietic Stem Cell Transplantation. *Eur. J. Drug Metab. Pharmacokinet.* **2019**, *44*, 659-668, doi:10.1007/s13318-019-00556-w.
12. Gastine, S.; Lehrnbecher, T.; Muller, C.; Farowski, F.; Bader, P.; Ullmann-Moskovits, J.; Cornely, O.A.; Groll, A.H.; Hempel, G. Pharmacokinetic Modeling of Voriconazole To Develop an Alternative Dosing Regimen in Children. *Antimicrob. Agents Chemother.* **2018**, *62*, doi:10.1128/aac.01194-17.
13. Liu, P.; Mould, D.R. Population pharmacokinetic analysis of voriconazole and anidulafungin in adult patients with invasive aspergillosis. *Antimicrob. Agents Chemother.* **2014**, *58*, 4718-4726, doi:10.1128/aac.02808-13.
14. Muto, C.; Shoji, S.; Tomono, Y.; Liu, P. Population pharmacokinetic analysis of voriconazole from a pharmacokinetic study with immunocompromised Japanese pediatric subjects. *Antimicrob. Agents Chemother.* **2015**, *59*, 3216-3223, doi:10.1128/aac.04993-14.
